# Supplementary material for: Meta-Analysis and Gene Set Enrichment Relative to ER Status Reveal Elevated Activity of MYC and E2F in the “Basal” Breast Cancer Subgroup
Source: PLoS One. 2009 Mar 9;4(3):e4710. doi: 10.1371/journal.pone.0004710 (PMC2650420; doi:10.1371/journal.pone.0004710)
Supplement: Dataset S1 — GSEA results from first screen. The file contains a zipped archive of the HTML results of the second GSEA screen. Reports for enriched or depleted datasets may be accessed from the “index.html” link within the my_analysis.GseaPreranked.1219975950625.rpt.zip ZIP archive. Enrichment plots are available for each of the 50 most enriched and depleted gene sets. (13.32 MB ZIP) [file pone.0004710.s001.zip › my_analysis.GseaPreranked.1219975950625.rpt.zip!//P21_P53_MIDDLE_DN.html]

Details for gene set P21\_P53\_MIDDLE\_DN[GSEA]

|  || Dataset | zSco.ERneg.to.ERpos\_collapsed |
| Phenotype | NoPhenotypeAvailable |
| Upregulated in class | na\_pos |
| GeneSet | P21\_P53\_MIDDLE\_DN |
| Enrichment Score (ES) | 0.7824107 |
| Normalized Enrichment Score (NES) | 2.4108016 |
| Nominal p-value | 0.0 |
| FDR q-value | 0.0 |
| FWER p-Value | 0.0 |
Table: GSEA Results Summary

  

Fig 1: Enrichment plot: P21\_P53\_MIDDLE\_DN      
 Profile of the Running ES Score & Positions of GeneSet Members on the Rank Ordered List

  

| PROBE | GENE SYMBOL | GENE\_TITLE | RANK IN GENE LIST | RANK METRIC SCORE | RUNNING ES | CORE ENRICHMENT || 1 | BUB1 |  |  | 41 | 6.445 | 0.0769 | Yes |
| 2 | AURKB |  |  | 76 | 6.038 | 0.1493 | Yes |
| 3 | MCM4 |  |  | 180 | 5.436 | 0.2090 | Yes |
| 4 | EXO1 |  |  | 218 | 5.239 | 0.2713 | Yes |
| 5 | CEP55 |  |  | 279 | 4.995 | 0.3288 | Yes |
| 6 | MCM7 |  |  | 289 | 4.970 | 0.3898 | Yes |
| 7 | CDC25B |  |  | 340 | 4.826 | 0.4459 | Yes |
| 8 | ASPM |  |  | 359 | 4.767 | 0.5037 | Yes |
| 9 | SMC2 |  |  | 651 | 4.028 | 0.5318 | Yes |
| 10 | MCM3 |  |  | 656 | 4.019 | 0.5814 | Yes |
| 11 | NCAPD3 |  |  | 661 | 4.016 | 0.6310 | Yes |
| 12 | CCDC99 |  |  | 737 | 3.863 | 0.6733 | Yes |
| 13 | TYMS |  |  | 829 | 3.738 | 0.7128 | Yes |
| 14 | TUBB3 |  |  | 1109 | 3.350 | 0.7334 | Yes |
| 15 | ATAD2 |  |  | 1532 | 2.917 | 0.7379 | Yes |
| 16 | WDHD1 |  |  | 1576 | 2.872 | 0.7703 | Yes |
| 17 | PBK |  |  | 1853 | 2.647 | 0.7824 | Yes |
| 18 | KNTC1 |  |  | 3562 | 1.679 | 0.6748 | No |
| 19 | HMGB2 |  |  | 3883 | 1.558 | 0.6701 | No |
| 20 | VEGF |  |  | 3911 | 1.549 | 0.6873 | No |
| 21 | TUBB4 |  |  | 5352 | 1.025 | 0.5917 | No |
| 22 | TUBA3 |  |  | 8675 | -0.160 | 0.3439 | No |
| 23 | ZNF84 |  |  | 9244 | -0.429 | 0.3065 | No |
Table: GSEA details [plain text format]

  

Fig 2: P21\_P53\_MIDDLE\_DN: Random ES distribution      
 Gene set null distribution of ES for **P21\_P53\_MIDDLE\_DN**
